# Association between constitution, axiography, analysis of dental casts, and postural control in women aged between 41 and 50 years

**DOI:** 10.1007/s00784-020-03571-3

**Published:** 2020-09-28

**Authors:** Daniela Ohlendorf, V. Fay, I. Avaniadi, C. Erbe, E. M. Wanke, D. A. Groneberg

**Affiliations:** 1 Institute of Occupational Medicine, Social Medicine and Environmental Medicine, Goethe-University Frankfurt am Main, Theodor-Stern-Kai 7, Building 9A, 60590 Frankfurt am Main, Germany; 2 Department of Orthodontics, School of Dentistry, University Medical Center of the Johannes Gutenberg University Mainz, Augustusplatz 2, 55131 Mainz, Germany

**Keywords:** Women, Axiography, Dental casts, Postural control, Constitution

## Abstract

**Objectives:**

The aim of this study was to investigate the relationship between anamnestic, axiographic and occlusal parameters and postural control in healthy women aged between 41 and 50 years.

**Materials and methods:**

A total of 100 female participants aged between 41 and 50 (45.12 ± 2.96) years participated in the study. In addition to completing a general anamnesis questionnaire, lower jaw movements were measured axiographically, dental occlusion parameters were determined using a model analysis and postural parameters were recorded using a pressure measurement platform. The significance level was 5%.

**Results:**

An increasing weight and a rising BMI lead to a weight shifted from the rearfoot (*p* ≤ 0.01/0.04) to the forefoot (*p* ≤ 0.01/0.02). A limited laterotrusion on the right resulted in a lower forefoot load and an increased rearfoot load (*p* ≤ 0.01). Laterotrusion to the left (extended above the standard) showed a lower frontal sway (*p* ≤ 0.02) and a reduced elliptical area, height and width (*p* ≤ 0.01, 0.02, 0.03). Thus, the extent of deviation correlated with reduced right forefoot loading (*p* ≤ 0.03) and the extent of deflection correlated with increased left foot loading (*p* ≤ 0.01). The higher the extent of angle class II malocclusion, the larger the ellipse area (*p* ≤ 0.04) and the ellipse height (*p* ≤ 0.02) resulted.

**Conclusions:**

There is a connection between weight, BMI and laterotrusion, as well as between angle class II malocclusion and postural control in women aged between 41 and 50 years. Interdisciplinary functional examinations of mandibular movements treating possible limitations can be conducive for an improvement of postural control.

**Clinical relevance:**

Angle class II malocclusion has a negative influence on postural control.

## Introduction

Control over posture and balance is an essential skill that is indispensable in everyday life and highly relevant to almost any movement or action. An upright posture can only be guaranteed by a muscular balance [1–6]. This is achieved by a balanced interaction of muscles and their counterparts and can compensate for possible disruptive factors [3, 5–7]. Postural control thus forms a complex interaction in the organism on many neuronal and sensomotoric levels [1, 3, 8]. Amongst others, a central role is attributed to trigeminal afferences which contain information from the muscle spindles of the masticatory muscles [9–11]. Therefore, a connection between postural control and the temporo-mandibular system can be assumed [5, 9, 11]. Whether an interference actually exists has been discussed for some time in literature [5, 11–19]; in some studies, correlations could be verified which proved changes in the postural control by different occlusion positions [5, 12, 13]. Accordingly, Ohlendorf et al. [12] found a reduced frontal and sagittal extension of the body’s centre of gravity by blocking the occlusion in contrast to the resting position. Hellmann et al. [20] succeeded in demonstrating that postural control is influenced by various motor tasks of the jaw, such as resting position, maximum biting on bilaterally placed cotton rolls or one-sided chewing. The results showed a statistically significant reduction of the elliptical area during force-controlled biting in contrast to the resting position. On the other hand, some studies [15, 17, 18] do not see any influence of different mandibular positions on postural control; this can be explained by compensation mechanisms covering changes in the temporo-mandibular system as well as trigeminal proprioception, for example. Thus, Tardieu et al. [17] could not demonstrate a difference in postural parameters between mandibular rest, maximum intercuspidation and simulated malocclusion on a stable base. Perinetti [15] also found no significant relationship between different mandibular positions and postural control.

Possible correlations between dental parameters and postural control are also discussed in literature [13, 14, 16, 19, 21, 22]. Accordingly, Perinetti et al. [16] found some weak correlations between malocclusions and postural control; the overbite showed correlations with anterior–posterior and lateral fluctuations [16]. Isaia et al. [19] found no correlation between dental parameters, such as angle class, overbite, overjet, crossbite, deviation and postural control in young and middle-aged subjects (23–44 years), which were investigated in static and dynamic conditions.

Regarding the dynamic movements of the mandible, Ohlendorf et al. [23] demonstrated a changed laterotrusion in subjects with hip arthritis in contrast to the control group. Accordingly, for the left laterotrusion, lower mean values exist for the limit values in the transverse plane in the left and right temporomandibular joint than in the control group. With regard to laterotrusion on the right side, higher values in the sagittal plane were found in the control group in the left temporomandibular joint. This demonstrates an effect of posture on mandibular movements. In another study, Heil et al. [24] also found a weakly significant change in movement during protrusion in subjects before and after total knee arthroplasty in contrast to the control group.

Therefore, the aim of this study was to investigate whether there exist correlations between the parameters of postural control and anamnestic, axiographic and dental parameters in women aged 41–50 years. The hypotheses were as follows:

1. The mobility of the lower jaw, and thus the extent of laterotrusion and protrusion movements in particular, is associated with changes in the load distribution of the forefoot and rearfoot.
2. There is a connection between a transverse deviation of the dental arches and percentage plantar load differences between the right and left foot.
3. Dental anomalies (angle classes, cross bite, overjet) are associated with fluctuations in the frontal and sagittal plane.

## Material and methods

### Subjects

In this study, 100 healthy female subjects aged 41 to 50 (45.12 ± 2.96) years with an average BMI of 25.36 ± 5.24 kg/m^2^ were enrolled without ongoing anamnesis. “Healthy” means that the subjects have no acute symptoms and subjectively described themselves as healthy at the time of measurement. According to the WHO classification [25], 60 subjects were of normal weight, 20 preadiposed and 18 obese. One person with a BMI below 18.4 kg/m^2^ was classified as being underweight.

At the beginning, all participants completed a medical history questionnaire of the Center for Dental, Oral and Maxillofacial Medicine of the Goethe University Frankfurt am Main (Germany) [26] regarding general diseases, such as diabetes mellitus, tinnitus, osteoporosis or rheumatism. Questions were also asked about pain in joints, in the musculoskeletal system, headaches and migraine, noises in the temporomandibular joint accidents and operations in the musculoskeletal system and medication. Furthermore, a short clinical examination in the sense of palpation was conducted with the test persons who reported occasional noises or pain in the temporomandibular joint. On the basis of this information, it was considered whether the existence of a temporo-mandibular disorder is likely, which is a strict exclusion criterion. Information about possible orthodontic treatments and sports activities was also included in the questionnaire; about 53% of the participants regularly engaged in sports, whilst the remaining 47% did not.

Further exclusion criteria for participation in this study were acute complaints or even injuries of the musculoskeletal system or the temporo-mandibular system, intake of muscle relaxants, medically diagnosed physical malpositions and current physiotherapeutic or orthopaedic therapies.

Due to non-evaluable measured values of one study participant, her data were not integrated into the evaluation.

The rights of these subjects were protected, and they were thoroughly familiarized with the study design before giving written informed consent to participate in this study. This study was approved by the local ethics committee of the medical faculty of the Goethe-University (Nr. 103/16) in accordance with the 1964 Helsinki Declaration and its later amendments.

#### Measurement systems

##### Axiography

The jaw registration system Jaw Motion Analyzer (Zebris Medical GmbH, Isny, Germany) is a measuring device with which function-analytical examinations can be carried out in the temporo-mandibular apparatus. The data acquisition is based on a radiation-free, ultrasound-supported system. According to the manufacturer, the JMAnalyser records the lower jaw mobility via a measuring sensor which contains four markers having a radiation and opening angle of 180° and which operate in a frequency range of 50 Hz. The measuring error is given as 0.1–0.2 mm [27–29]. The measuring system consists of a face bow with receiver modules, a lower jaw/pointer sensor, a basic unit with power supply, a wireless footswitch and the attachment. To register the jaw movements, an attachment must first be fixed to the lower teeth row; this was achieved with a bite registration material (“Luxabite,” DMG Dental Material GmbH, Hamburg, Germany). The measuring system is connected to a computer via USB. With the corresponding WINJAW+ software, motion and function analyses can be evaluated.

##### Posturography

The postural control was determined by using the pressure measuring platform GP MultiSens (GeBioM GmbH, Münster, Germany). This sensor plate has a measuring area of 38.5 cm by 38.5 cm and contains 2304 matrix-shaped sensors. The size of a sensor is about 8.8 mm. The measuring frequency is 100 Hz per sensor (a total sampling rate of approximately 500 kHz). The sensors are scanned by an internal USB measuring interface.

Plantar pressure distribution was detected by the matrix-shaped arrangement and a high-impedance gain and transferred to software via the USB connection. The measuring error is ± 5%. The GPManager programme is used as evaluation software.

##### Impression and orthodontic model analysis

To perform a model analysis, impressions were taken using alginate (Trealgin Chromatic, Schütz Dental group, Rosbach von der Höhe, Germany) in the maxilla and mandible. Using a modelling wax (modelling wax standard 175/80, 1.25 mm, Gebrüder Steinhart Wachswarenfabrik GmbH & Co. KG), a bite registration was carried out. The dental casts were produced with hard plaster (Natura DIN EN ISO 6873 Type 3, Siladent Dr. Böhme & Schöps GmbH, Goslar).

#### Medical history questionnaire

The medical history questionnaire of the Centre for Dental, Oral and Maxillofacial Medicine of the Goethe University Frankfurt am Main [26] was used and includes questions about the following: allergies, osteoporosis, rheumatism, diabetes, tinnitus, neurological diseases, headache/migraine, pain in joints, pain/sound in the temporomandibular joint, pain in the back, accidents on the face/accidents on shoulders and/or back and/or pelvis, previous operations, orthopaedic therapy, regular drug intake, orthodontic therapy or sporting activity.

#### Examination procedure

When conducting the measurements, all test subjects undergo the axiography, the posturographic measurement and the impression of the dental casts one after the other:

##### Axiography

For the position analysis of the mandible, a pre-bent attachment had to be fixed buccally to the mandibular row using Luxabite (DMG Dental Material GmbH, Hamburg, Germany). The attachment must be stable and resist movements including the process of attaching the receiver module without interfering with static and dynamic occlusion. After adjusting the facebow, the receiver module was calibrated and fixed to the attachment. The measurements were performed with the eyes open, and each subject was asked to focus their gaze straight ahead in the direction of vision at eye level.

With the “Function” module of the corresponding WINJAW+ software, various jaw movements were performed within the physiological boundary space. The lower jaw movements were measured three times in succession. The following parameters were recorded: maximum mouth opening (mm), maximum protrusion (mm), maximum laterotrusion to the right and left (mm) and the deviation and deflection of the mandible (mm). Depending on the length of the laterotrusion path, a division into a normal range group (7–12 mm), hypomobility (< 7 mm) and hypermobility (12 > mm) groups was performed [30].

##### Posturography

For posturography, the subjects were placed on the pressure measuring plate without footwear and positioned into the habitual posture to be adopted; the arms loosely suspended from the body and the lower jaw positioned in a resting position and looking straight ahead in the direction of vision at eye level with open eyes. The subjects were asked to remain in this position during the entire measurement without speaking or moving. In this position, three measurements were performed for 30 s per subject.

The load distributions of the entire right and left foot, as well as the forefoot and rearfoot, right and left, plus the entire forefoot and rearfoot, are presented as a percentage. Frontal and sagittal sway were recorded in mm. Further parameters to be measured were the elliptical area with the unit cm^2^, the area of which is composed of the fluctuation of movement in the area of the centre of gravity, the height of the ellipse (cm), the width of the ellipse (cm) and the angle (°).

##### Dental casts and model analysis

The impressions were taken with alginate in the upper and lower jaw and the dental casts were subsequently fabricated with hard plaster. The dental casts were used for evaluation and in a simplified model analysis according to the Frankfurt principle. The following parameters had to be determined: midline shifts, overjet, overbite, crossbite, Angle class molar relationsships and transverse width difference.

The study participants were divided into groups according to the parameters of the model analysis.

##### Midline shifts

Deviations from the midline were given in mm in both the upper and lower jaw and then divided into three groups according to direction: no deviation, deviation to the left and deviation to the right. In the upper jaw, the number of a midline shift to the left was only 8; due to the small number, this parameter was taken from the evaluation.

##### Transversal width

The transverse width was measured in mm and compared with the nominal value. The target value is taken from the target value table from “Curriculum Kieferorthopädie” [31]. The difference was given in mm.

##### Occlusion

The occlusion was divided into neutral (angle class I), distal (angle class II) and mesial (angle class III) occlusion. The respective distal or mesial deviation was indicated in mm.

##### Overjet/overbite

Overjet and overbite were measured in mm.

##### Cross bite/edge-to-edge bite/buccal occlusion

A division into 2 groups was made in each case, whether a cross, edge-to-edge bite or buccal occlusion was present or not.

Due to the small number of samples, the following parameters were not considered in the evaluation: cross bite on the left (*n* = 9) and buccal occlusion on the right (*n* = 2) and left (*n* = 0).

##### Statistical analysis

The collected values were evaluated with the statistic programme BiAS 11.10 (epsilon-Verlag, Darmstadt, Germany). Initially, all parameters were tested for normal distribution using the Kolmogorov-Smirnov test. According to the distribution, the following tests were used: the Wilcoxon-Mann-Whitney *U* test, two-sample *t* test and the Kruskal-Wallis test with post-doc-tests (Conover-Iman comparison). All *p* values undercut the Bonferroni-Holm correction.

For correlations, the Spearman and Kendall rank correlation test for non-normally distributed data and the Pearson simple linear regression test for normally distributed data were applied.

The significance level for all tests was set to a *p* value of less than 0.05. The *p* value for all tests was 0.05. The effect strength serves to evaluate the correlation coefficient rho according to Evans and is defined as follows: 1 = < 0.2: poor; 2 = 0.2–0.4: weak; 3 = 0.4–0.6: moderate; 4 = 0.6–0.8: strong; 5 = > 0.8: optimal.

## Results

### Anamnestic parameters

**Fig. 1:**
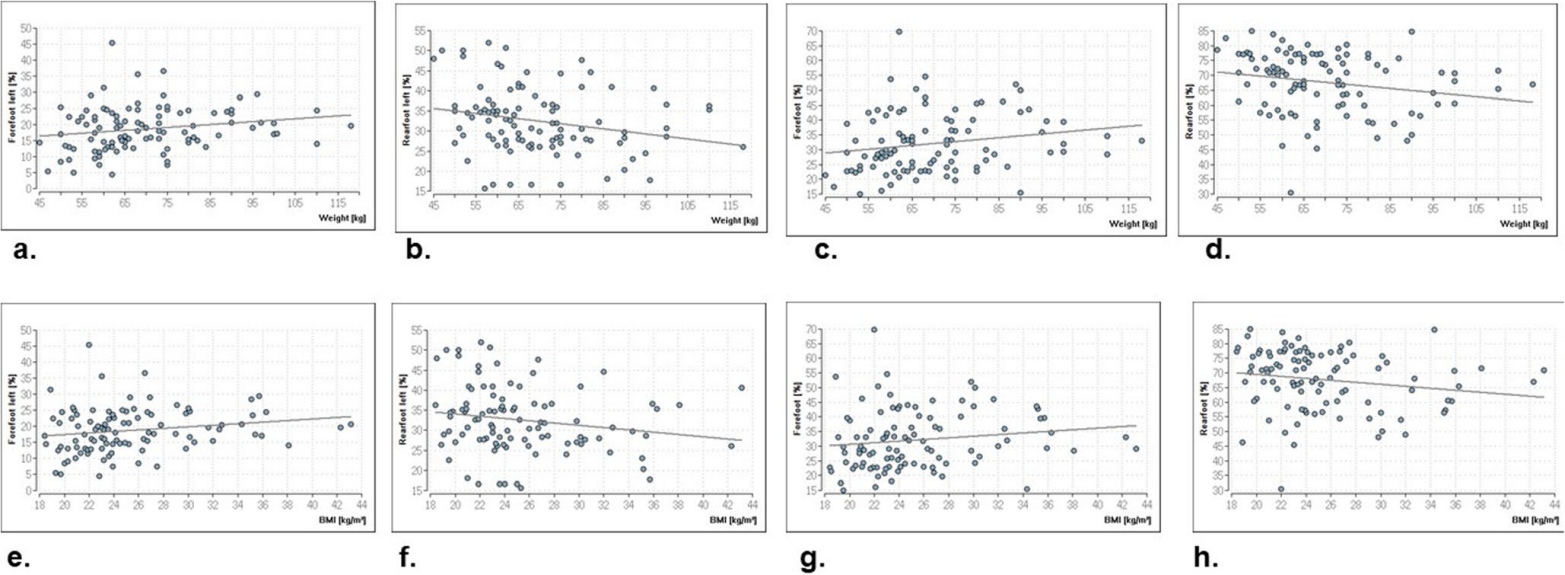
Significant correlations between the load parameter and the weight/BMI. The positive correlation illustrates that with increasing weight there is a higher load on the left forefoot (**a**). The load distribution of the left forefoot decreases with increasing weight (**b**). An increasing weight is accompanied by a higher load on the entire forefoot and a lower load on the entire rearfoot (**c**, **d**). The higher the BMI, the greater the load is found on the left forefoot (**e**) and the entire forefoot (**f**), whilst a lower load is found on the left (**g**) and the entire rear foot (**h**)

The correlations of the demographic parameters (height, weight and BMI) prove that body height has no significant influence on postural control whereas weight and body mass index show significance for the load on the left forefoot (*p* ≤ 0.02 and 0.01, respectively), the left rearfoot (*p* ≤ 0.01 and 0.04, respectively) and the entire forefoot (*p* ≤ 0.01 and 0.02, respectively) and rearfoot (*p* ≤ 0.01) (Fig. 1a–1h). The effect strength is weak or poor. For all other parameters, there are no significances (*p* ≥ 0.05).

**Fig. 2:**
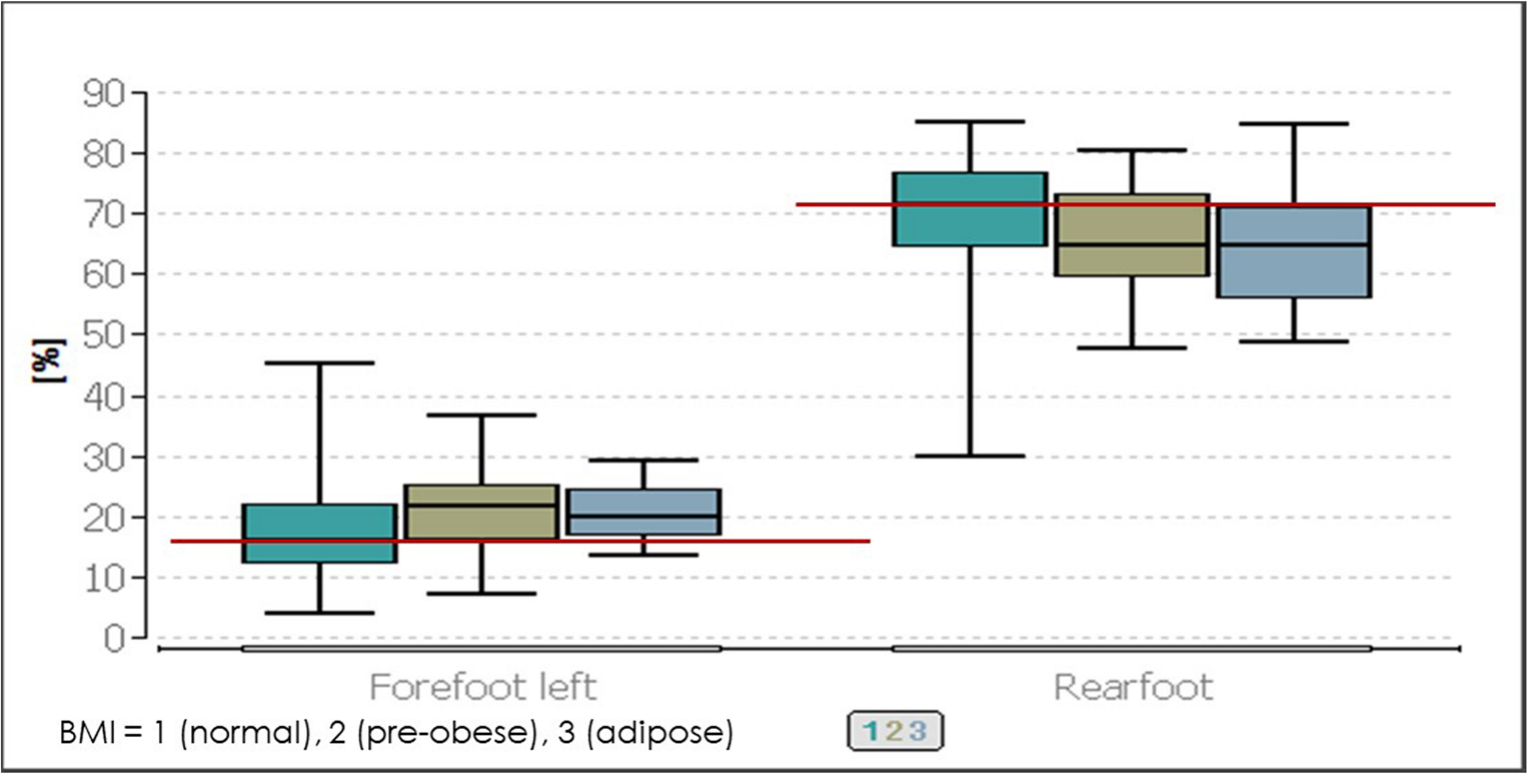
Comparison of the postural parameters forefoot left and rearfoot in the respective BMI classes (normal, pre-obese, obese). The lower red line represents the median value of the forefoot left at BMI 1 (normal), and the upper red line represents the median value of the rearfoot at BMI 1 (normal)

When the three different BMI classifications (normal, pre-adipose and obese) are compared with each other with the parameters of the postural control, significances can be observed in the load distribution of the left forefoot (*p* ≤ 0.01) and the entire rearfoot (*p* ≤ 0.04). All other parameters show no significance (*p* ≥ 0.05). The following Conover-Iman test showed, with *p* ≤ 0.04 significances between the group of the “normal” BMI versus the “pre-obese” group regarding the left forefoot load, that they differ in the median value by 4%. The comparisons of “normal” versus “obese” and “pre-obese” versus “obese” are also significant with *p* values of *p* ≤ 0.05. The total load on the rearfoot showed exclusively a global significance (Fig. 2).

### Mandibular movements

Table 1 gives an overview of the extent of the laterotrusion to the left and right, as well as the protrusion and opening movement. The laterotrusion to the right was 9.37 ± 2.44 mm on average and 9.95 ± 2.16 mm to the left. The length of the protrusion line was 9.90 ± 1.91 mm. The mouth opening had an average value of 43.54 ± 6.3 mm.

**Table 1:** Mean values and extent of mandibular movements

	Mean value ± SD	Minimum	Maximum
Laterotrusion to the right (mm)	9.37 ± 2.44	3.00	14.00
Laterotrusion tot the left (mm)	9.95 ± 2.16	4.00	15.00
Protrusion (mm)	9.90 ± 1.91	4.00	16.00
Mouth opening (mm)	43.54 ± 6.3	22.00	56.50

### Laterotrusion

For the laterotrusion, correlation tests were performed on the one hand, whilst, on the other, the individual groups were compared; these were set up according to the length of the laterotrusive path (see the “Axiography” section).

The extent of a laterotrusion to the right or to the left was not found to correlate with the postural parameters.

In contrast, significance can be demonstrated for the group comparisons with respect to the laterotrusion to the right (Table 2). The groups were divided according to the length of the laterotrusion path and, thus, the mobility both on the right and on the left side. Significant group differences can be found with *p* ≤ 0.04 and 0.01, respectively, in the forefoot, hindfoot and forefoot right parameters. For all other parameters there are no significances (*p* ≥ 0.05).

**Table 2:** Median, minimum, and maximum of posturographic parameters for the extent of lower jaw movements regarding the laterotrusion right and left. Comparison between movements in the normal range, hypomobility, and hypermobility of the mandible. Significant *p* values are printed in bold

Laterotrusion to the right							
	Normal range (7–12 mm) *n* = 72		Hypomobility/below normal range *n* = 14		Hypermobility/above normal range *n* = 13		*p* value
	Median	Min./max.	Median	Min./max.	Median	Min./max.	
Forefoot left (%)	17.67	4.33/45.33	23.00	10.67/35.67	16.00	7.33/31.33	0.15
Forefoot right (%)	12.34	2.50/29.67	19.33	7.00/26.33	12.33	2.00/22.67	**0.04**
Rearfoot left (%)	32.67	15.67/52.00	27.84	16.67/40.33	29.00	22.50/44.33	0.05
Rearfoot right (%)	35.00	2.67/55.33	36.00	14.67/46.67	37.00	19.67/63.00	0.72
Leftfoot (%)	51.78	28.33/73.00	50.17	38.67/66.67	50.00	35.00/60.00	0.34
Rightfoot (%)	48.22	27.00/71.67	49.84	33.33/61.33	50.00	40.00/65.00	0.33
Forefoot (%)	28.50	15.33/69.67	40.00	27.67/54.67	33.00	15.00/53.67	**0.01**
Rearfoot (%)	71.50	30.33/84.67	60.00	45.33/72.33	67.00	46.33/85.00	**0.01**
Frontal sway (mm)	13.00	4.00/38.00	12.67	7.33/26.67	14.00	6.00/25.50	0.10
Sagittal sway (mm)	16.50	1.50/43.67	19.50	6.33/26.67	15.33	2.33/25.67	0.49
Elliptical surface (cm^2^)	1.35	0.06/8.79	1.73	0.29/3.38	1.16	0.23/4.20	0.56
Ellipse width (cm)	0.98	0.26/2.26	1.15	0.49/.32	0.85	0.46/1.74	0.82
Elliptical height (cm)	0.44	0.05/1.28	0.49	0.18/0.80	0.46	0.10/6.49	0.65
Angle (°)	− 23.68	− 87.16/73.11	− 8.52	− 52.08/58.50	− 27.05	− 79.93/86.08	0.30
Laterotrusion to the left							
	Normal range (7–12 mm) *n* = 76		Hypomobility/below normal range *n* = 10		Hypermobility/above normal range *n* = 13		*p* value
	Median	Min./max.	Median	Min./max.	Median	Min./max.	
Forefoot left (%)	19.00	4.33/45.33	21.17	9.00/28.33	16.00	7.33/31.33	0.60
Forefoot right (%)	12.00	2.00/25.67	19.17	10.44/28.00	18.33	3.33/29.67	**0.01**
Rearfoot left (%)	32.17	15.67/52.00	28.95	16.67/50.00	28.67	26.33/36.00	0.53
Rearfoot right (%)	35.33	2.67/63.00	27.34	21.00/44.11	36.33	19.67/51.67	0.13
Left foot (%)	52.11	28.33/73.00	51.17	38.67/60.33	49.33	35.00/60.00	0.26
Right foot (%)	47.89	27.00/71.67	48.50	39.67/61.33	50.67	40.00/65.00	0.25
Forefoot(%)	29.00	15.00/69.67	42.00	23.00/52.00	33.00	19.67/53.67	0.07
Rearfoot (%)	71.00	30.33/85.00	58.00	48.00/77.00	67.00	46.33/80.33	0.09
Frontal sway (mm)	14.33	5.00/27.33	11.33	9.00/22.33	11.00	4.00/16.00	**0.02**
Sagittal sway (mm)	17.42	1.50/27.67	19.00	6.33/28.33	11.00	2.33/23.33	0.06
Elliptical surface (cm^2^)	1.49	0.06/4.43	1.38	0.29/3.43	0.77	0.23/2.47	**0.01**
Ellipse width (cm)	1.06	0.43/2.02	1.00	0.26/2.28	0.85	0.42/1.31	**0.02**
Elliptical height (cm)	0.46	0.05/1.09	0.49	0.18/1.28	0.31	0.10/0.59	**0.03**
Angle (°)	− 18.99	− 87.16/73.11	− 16.61	− 52.08/54.07	− 24.63	− 84.01/86.08	0.89

**Fig. 3:**
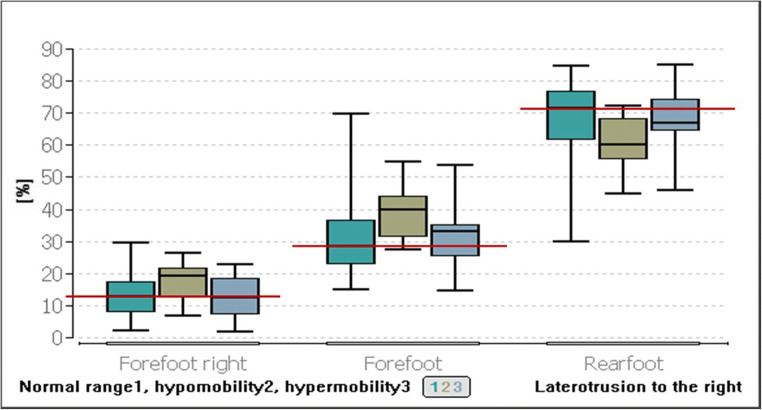
Comparison of postural parameters forefoot right, total forefoot, and total rearfoot with different extents of movement of the right laterotrusion

After Bonferroni-Holm correction, the Conover-Iman test showed a significance of *p* ≤ 0.04 between the group in the normal range and the group of hypomobility with a difference of 6.99% in the median value of the right forefoot. Laterotrusion to the right results in a low load on the right forefoot in the normal range compared with laterotrusion below the normal range (Fig. 3).

With regard to the loading of the entire forefoot, there is also a significance between the groups normal range of the right laterotrusion and the laterotrusion (right) below the normal range (*p* ≤ 0.01). The load on the forefoot was found to be 11.5% lower in women with a laterotrusion in the normal range than in women with a laterotrusion below the normal range (Fig. 3).

For the loading of the entire rearfoot, the significance concerns the two groups of laterotrusion in the normal range and laterotrusion below the normal range (*p* ≤ 0.01). Women with a laterotrusion to the right in the normal range were found to load their rearfoot median by 11.5% more than women with hypomobility to the right laterotrusion (Fig. 3).

In the following, the comparisons of the laterotrusion to the left were divided into groups with a movement in the normal range, the hypomobility range or the hypermobility range (Table 2).

Statistical significances were found in the load on the right forefoot (*p* ≤ 0.01), the frontal sway (*p* ≤ 0.02), the elliptical surface (*p* ≤ 0.01), the elliptical width (*p* ≤ 0.02) and the elliptical height (*p* ≤ 0.03). For all other parameters, there were no significances (*p* ≥ 0.05).

The Conover-Iman test showed that a significance (*p* ≤ 0.02) was observed for the right forefoot load between the groups with a laterotrusion in the normal range and hypomobility. The study participants, whose laterotrusion to the left was within the normal range, loaded their right forefoot by 7.17% median less than those with hypomobility.

**Fig. 4:**
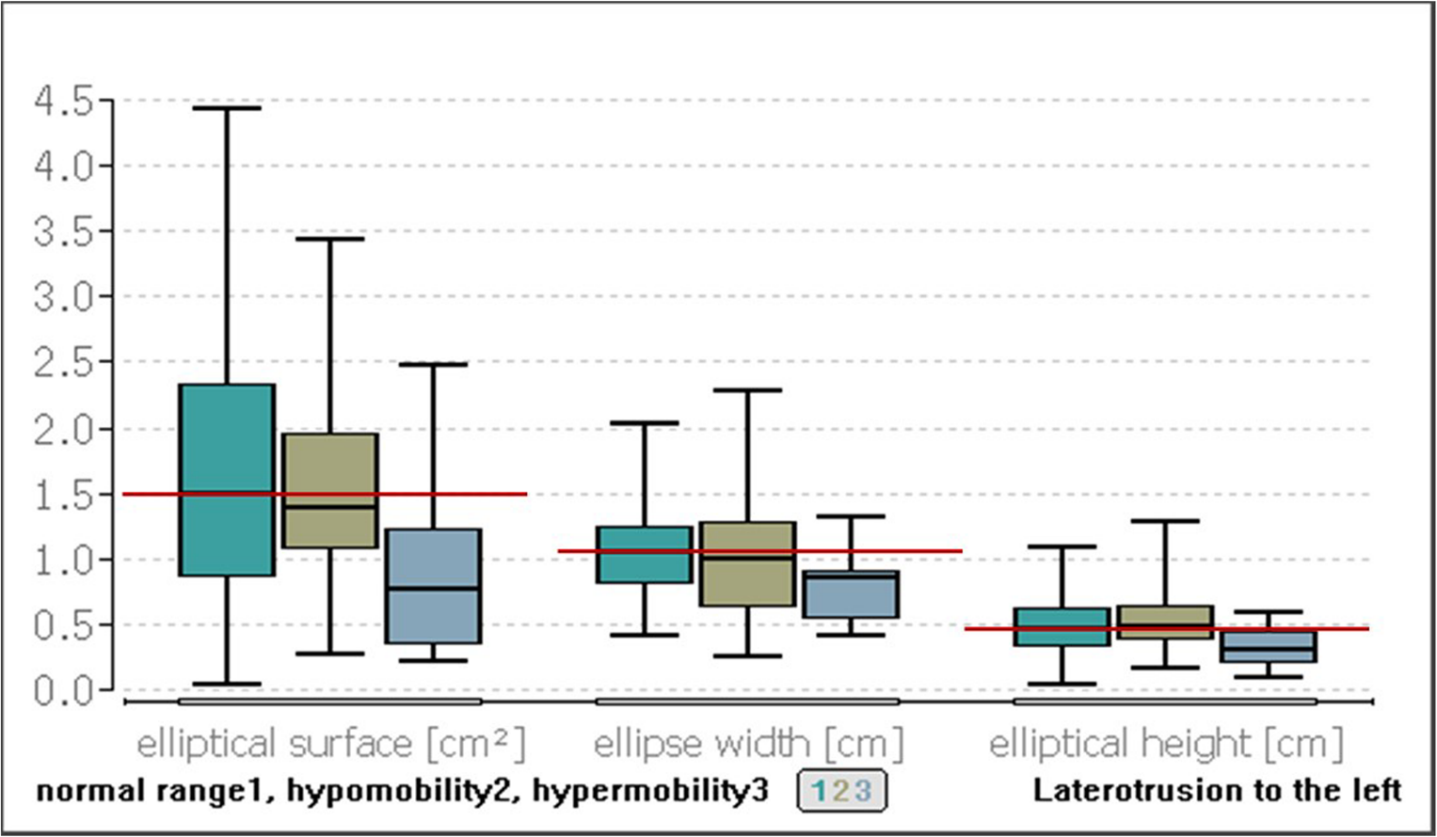
Comparison of the postural parameters for the elliptical surface, ellipse width, and elliptical height with different degrees of movement of the left laterotrusion. The red line represents the median value for the group in the normal range

The difference between the elliptical area of the study participants in the normal range and that of the study participants with hypermobility of the left laterotrusion was found to be 0.72 cm^2^ (*p* ≤ 0.01, Fig. 4). The width of the ellipse was found to differ significantly in its median values by 0.21 cm (*p* ≤ 0.01) between the group with the laterotrusion left in the normal range and the group with the hypermobility (Fig. 4).

The median value of the ellipse height in the group of the normal range was 0.46 cm and that of the group of hypermobility was 0.31 cm; thus, a difference of 0.15 cm was calculated for the height of the ellipse (*p* ≤ 0.04, Fig. 4).

**Fig. 5:**
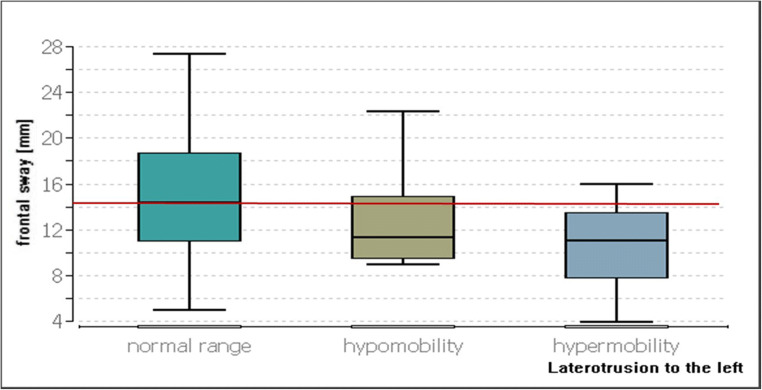
Comparison of the postural parameters for the frontal sway with different degrees of movement of the left laterotrusion. The red line represents the median value of the group in the normal range

For the frontal sway, the hypermobility group was found to have a 3.33 mm lower sway than the group in the normal range (*p* ≤ 0.03, Fig. 5).

### Mouth opening/protrusion/deviation/deflection

There were no statistically significant *p* values with regard to a correlation between the extent of the mouth opening or protrusion and the postural parameters.

**Fig. 6:**
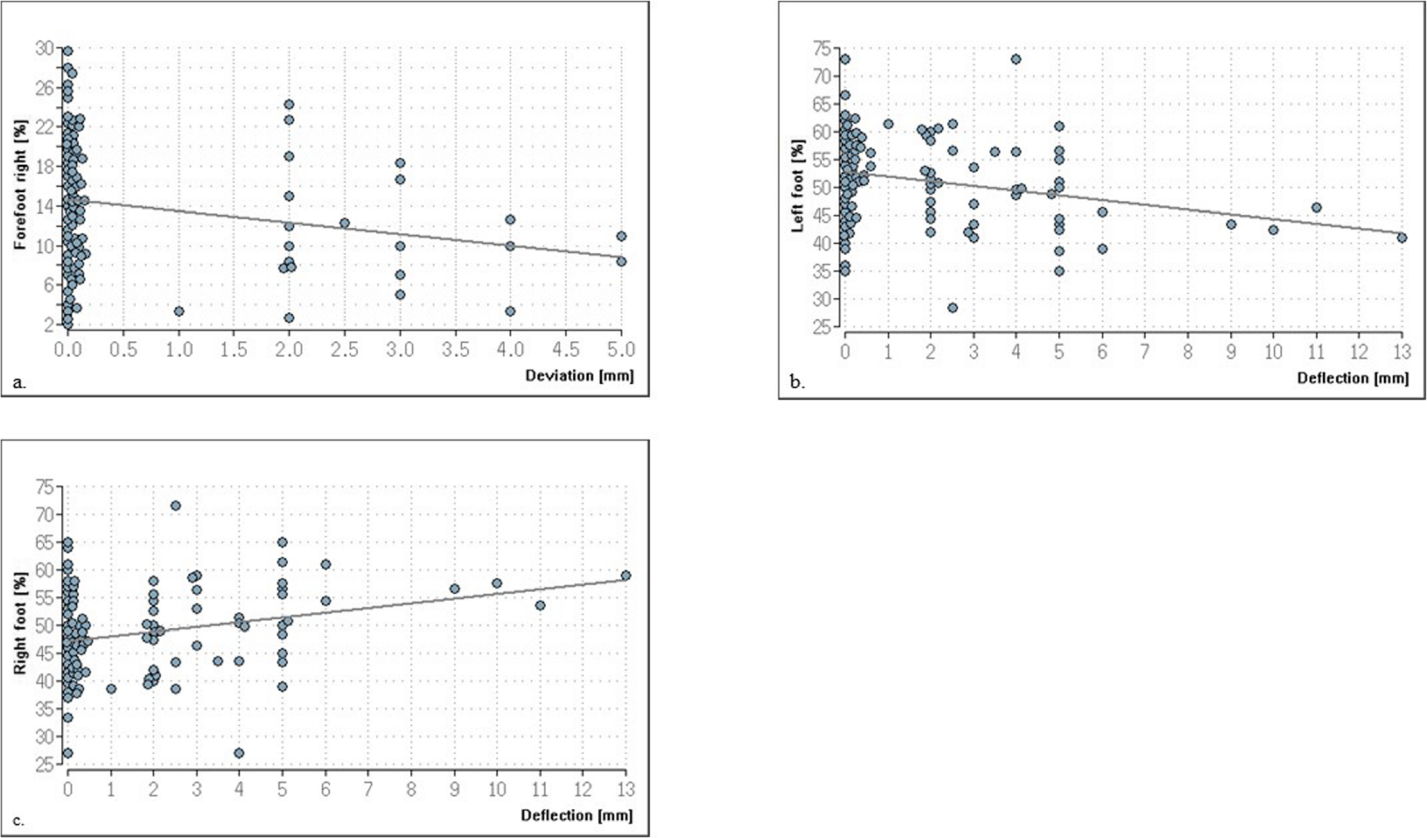
Significant correlations between the load of the right forefoot or the left and right entire foot. (**a**) Illustrates the higher the extent of deviation, the lower the load on the right forefoot results. (**b**) Illustrates the correlation that the lower the deflection, the higher the load on the left foot results. Similarly, the correlation between deflection and the load on the right foot (**c**) shows that the greater the deflection, the greater the load on the right foot becomes

There is one significant correlation between the extent of deviation and the percentage load on the right forefoot (*p* ≤ 0.03, rho: − 0.22, Fig. 6a) and the deflection and percentage load on the left foot and right foot (*p* ≤ 0.01; rho left: − 0.26, rho right: 0.26, Fig. 6b+c).

The comparison between deflection/deviation to the right and left and a regular mouth opening without deviation related to the posturographic parameters do not give significant data values for the load distribution when comparing all three groups with each other.

### Model analysis

There were no significant correlations (*p* ≥ 0.05) for the postural parameters and the extent of a midline shift in both the mandible and the maxilla. There were also no statistically significant correlations for the postural control and the extent of a transverse width difference between the actual and target value.

Below, the results of the influence of occlusal parameters on postural control are presented. A total of 51 of the study participants had an angle class I molar relationship on the right side and 38 on the left side. An angle class II molar relationship was present in 34 of the study participants on the right and 37 on the left. The proportion of an angle class III molar relationship was found to be the lowest with 14 of the study participants on the right and 24 on the left.

Table 3 shows that the extent of distal occlusion in the left affects the ellipse surface (*p* ≥ 0.04; rho: 0.35; effect strength “weak”).

**Table 3:** Correlation of the extent of distal occlusion with postural parameters. Significant *p* values are marked in bold

	Right			Left		
	*p* value	rho	Effect-size	*p* value	rho	Effect-size
Forefoot left (%)	0.60	0.09	Poor	0.58	− 0.1	Poor
Forefoot right (%)	0.54	− 0.11	Poor	0.35	0.16	Poor
Rearfoot left (%)	0.50	− 0.12	Poor	0.99	− 0.002	Poor
Rearfoot right (%)	0.79	− 0.05	Poor	0.60	0.09	Poor
Left foot (%)	0.91	0.02	Poor	0.69	− 0.07	Poor
Right foot (%)	0.91	− 0.02	Poor	0.69	0.07	Poor
Forefoot(%)	0.77	0.05	Poor	0.54	0.10	Poor
Rearfoot (%)	0.77	− 0.05	Poor	0.54	− 0.10	Poor
Frontal sway (mm)	0.13	0.27	Poor	0.99	− 0.002	Poor
Sagittal sway (mm)	0.05	0.36	Weak	0.05	0.32	Weak
Elliptical surface (cm^2^)	0.26	0.2	Weak	**0.04**	**0.35**	**Weak**
Ellipse width (cm)	0.13	0.26	Weak	0.17	0.23	Weak
Elliptical height (cm)	0.86	0.03	Weak	**0.02**	**0.38**	**Weak**
Angle (°)	0.50	− 0.12	Poor	0.11	0.27	Weak

The greater the extent of distal occlusion, the larger the ellipse area. There was also a correlation with the height of the ellipse (*p* ≥ 0.02, rho: 0.38, effect strength “weak”). There are no correlations for all other parameters (*p* ≥ 0.05). With regard to mesial occlusion, no statistically relevant effects on postural control were evident.

Overjet and overbite revealed no significant correlation with the parameters of posturography.

A crossbite was observed in 19 study participants on the right side and in 9 on the left side, whilst 7 study participants had a crossbite on both sides. 15 of the volunteers had an edge-to-edge bite on the right as well as on the left side and 7 of the volunteers had an edge-to-edge bite on both sides. Due to the small number of samples of a crossbite on the left side, this group was not integrated into the calculations, as was buccal occlusion.

Whether a crossbite is present on the right or not, this does not have a statistically significant effect on the postural stress parameters (*p* ≥ 0.05).

Similar to the crossbite, the presence of an edge-to-edge bite on both the right and left side was found to have no significant influence on the postural control (*p* ≥ 0.05).

## Discussion

The aim of this study was to investigate whether there is a correlation between postural control parameters and anamnestic, axiographic and dental parameters in a homogeneous age-group of 100 women aged between 41 and 50 years. The objective was to minimize age-specific and gender-specific influencing factors, such as differences in hormonal balance and body constitution [32, 33], in order to ensure good comparability of the test results. The 41–50 years age group represents an interesting group of female volunteers as they are exactly in their middle years [34], and their physical condition does not correspond to those of 20 to 30 year olds or mid to late 50 year olds. Women at this age are just pre-menopause. The onset of menopause is variably defined in different data sets and is subject to individual fluctuations [35–37]. In most cases, however, menopause does not occur until the beginning of the 50s [35]. Accordingly, the influence of hormonal changes in the present age group is not yet, or hardly, present. Nevertheless, the first ageing processes do become noticeable in this age group [38].

According to the WHO, between 30–80% of adults and about 30% of children in Europe are overweight (BMI above 25 kg/m^2^) [39]. In addition to cardiovascular diseases and the development of type 2 diabetes, being overweight also has an effect on body geometry and posture [32, 40]. For example, the position of the centre of gravity is adjusted and shifted anteriorly due to the excess weight in the abdominal region of overweight people; in addition, there is an increased ankle joint moment in order to maintain balance [40–42].

For the constitutional parameters, this study showed the same tendencies for both weight and the BMI for the collective. An increased weight and an increased BMI correlate with an increasing load on the forefoot and a decreasing load on the rearfoot. Heavier and overweight women shift their centre of gravity anteriorly when compared to women of normal weight. The detailed group comparison shows that pre-obese women put 5% more load on their left forefoot (21.84%) than normal-weight women (15.84%). An average forefoot and rearfoot load is 33 to 66% [43, 44]. The rearfoot load tends to be higher in the case of normal-weight women (71.67%) than in the case of pre-obese (64.84%) and obese women (64.67%).

Rezaeipour [40] raised the assumption that the anterior shift of the centre of gravity is due to the increase in weight in the abdominal area. This study reinforces their assumption since an increase in the weight of the breast, especially in overweight women, can be found. According to Menegoni et al. [32], overweight men and women show instability in the anterior–posterior region whilst overweight men also show a shift of the centre of gravity in the mediolateral region; the different body constitution or fat distribution between the sexes is the reason for this [45, 46]. In this study, however, no correlations could be observed with regard to sway and being overweight, which may be associated with a biomechanical adaptation of posture control in being overweight [47]. Nevertheless, it must be taken into account that the participants were not selected according to the corresponding BMI category; therefore, unequal group sizes were present which limits a clinically relevant statement.

The analysis of lower jaw movements is an important part of the diagnosis of the temporomandibular system. Accordingly, deviations in the motion sequence and extent of movement can lead to disturbances and dysfunctions in this area or be the result of dysfunctions in the masticatory system [48].

In this context, it must be taken into account that although diagnostic criteria for temporomandibular disorder (TMD) represent an improvement over RDC/TMD, their immediate implementation in research and health care does not yet seem sufficiently justified [49]. Therefore, we have decided to use the medical history questionnaire of the Center for Dental, Oral and Maxillofacial Medicine of the Goethe University Frankfurt am Main (Germany), which was designed, validated and evaluated by Kopp [26].

In this study, correlations between mandibular movements and postural control could be proven. In the case of laterotrusion to the right, the entire forefoot and, in particular, the right forefoot is loaded 11.5 and 6.99% less in subjects with a movement in the normal range than in those below the normal range; equivalent to this, more load was placed on the rear foot. Furthermore, in women with a greater extent of movement of the left lateral extrusion, a lower frontal sway and a smaller elliptical area, height and width could be determined. The median load on the right forefoot was 7.17% greater in women with laterotrusion in the normal range than in those with hypomobility. Presumably, a large range of motion of the mandible reflects a well-balanced and well-functioning muscle activity which indicates a balance without functional disturbances in the craniomandibular system. Therefore, it can be assumed that limited mandibular movement triggers an imbalance that may be reflected in postural control. Proven connections between disorders in the masticatory system and cervical spine posture, as well as the general posture, support this assumption [50, 51]. Although none of the subjects reported complaints in the masticatory system, restricted mandibular movements are a factor that can cause temporomandibular dysfunction [52]. Limitations of the mandible can be caused by an increase in collagen fibres; this can occur, for example, through scarring after tissue damage, through increased muscle tension or dislocations of the discus articularis [52]. Therefore, mandibular movements are measured in diagnostic procedures and classified according to severity [48, 53]. For this reason, the results presented here are compared with studies investigating correlations between postural control and TMD patients as the limitation of mandibular movements is often present in TMD patients [52]. Data on mandibular movements in healthy subjects and postural control are currently not available.

However, when interpreting the data with regard to the axiography, it must be taken into account that this measuring method was not familiar to the participants. Under these circumstances, movement artefacts or “out-of-round” movements may have been performed, which may not exactly correspond to the real movement and could give insight into the presence of a pathology. Since we cannot differentiate between patients who have real restrictions or whether this is due to an improper measurement, this should not be chosen as a definitive criterion for the evaluation of restrictions of the mouth opening or lateral movement.

In the present study, there were relatively small differences of 3.3 mm in the frontal sway and 0.72cm^2^ in the elliptical area and, therefore, these were clinically less relevant, however, these tendencies should not be neglected. Furthermore, changes in the weight distribution can be observed. The 1st hypothesis, which states that jaw mobility is associated with the percentage change in plantar foot pressure distribution, can be verified, but only small changes are detectable, which are hardly of clinical relevance. These correlations only apply to laterotrusion movements; no correlations could be established for protrusion and mouth opening. Nota et al. [8] confirmed the results obtained in this study since an increased fluctuation area could be demonstrated in subjects with TMD in comparison to the control group. On the contrary, Manfredini et al. [54] and Oltramari-Navarro et al. [55] presented the opinion that there are no measurable occlusal and postural abnormalities associated with craniomandibular dysfunctions.

Weakly significant correlations result for deviations and deflections in the entire left and right foot; the higher the extent of deviation, the lower the load on the right forefoot results and, the lower the deflection, the higher the load on the left foot becomes. Deviations of the lower jaw in opening movements can lead to a changed chewing pattern which, in turn, has effects on the craniomandibular function [56]. Wakano et al. [57] achieved deterioration of postural control on unstable ground by using a splint which forced an experimentally temporary deviation. The authors assume that the provoked occlusal interference is a supposed stressor that induces muscle tension that affects the maintenance of dynamic balance via anti-gravity muscle and ankle strategies. Due to the weak correlations, these results have little clinical relevance; however, present tendencies do prove the connection between the craniomandibular system and postural control [6, 9, 11, 14, 20].

The model-analytical evaluation of the dental casts revealed only a few significant correlations with postural control. Midline shifts were more frequent in the lower jaw (63%) than in the upper jaw (23%). In general, deviations of the midline can affect the entire occlusion and the temporomandibular joint [58]; however, no effects on postural control were observed in this study by these deviations. With regard to the transversal width difference and crossbite, no measurable effects could be observed in the postural parameters. A discrepancy of the transverse width from the nominal value does not necessarily have to be accompanied by an irregular occlusion or muscular imbalance; this could be indicated by a crossbite or a forced bite. The body has the potential to adapt to this permanent dysbalance over a long period of time so that no differences in postural control can be detected. Therefore, the second hypothesis, which states that there is a connection between a transverse deviation of the dental arches and percentage plantar load differences between the right and left foot must be falsified.

In contrast, there are weak correlations between the molar relationship and postural control: the stronger the degree of distal occlusion (angle class II), the larger the elliptical area and height become. Malocclusions are associated with functional changes, ranging from chewing and jaw joint dysfunction to changes in the neck and spine [59]. To compensate for this, the body probably needs to do more work on the compensation mechanisms which produces a negative effect on postural control. Opposing to angle class II malocclusion subjects, no significance was found for subjects with mesial occlusion (angle class III). It should be mentioned, however, that the number of class III subjects was significantly smaller, which also reflects the current data with regard to the prevalence of malocclusions. [60]

Crossbites and edge-to-edge bites were observed only in a small number of subjects. Although these malocclusions lead to forced bites and can, therefore, be associated with functional disorders [31], no significant correlations with postural control could be demonstrated in this study. This was confirmed by further studies in children and young adults [61], as well as in adults, by comparisons with their respective control groups [21].

In middle-aged women, dental anomalies, therefore, have no significant influence on postural control. The third hypothesis, which states that dental anomalies are associated with fluctuations in the frontal and sagittal plane cannot be verified. Significances can be recorded for individual parameters with angle class II malocclusion playing a role. The craniomandibular system can generate compensations which may initially be limited to the head/neck area, but this altered head posture consequently results in causing small imbalances in posture [16, 19]. Scharnweber et al. [13] also refuted a connection between dental malpositions and postural control in men (18–35 years) and explained this by compensation mechanisms of the body. In contrast, Cuccia [14] postulates that there is a detectable relationship between the craniomandibular system and postural control which is clinically relevant. Accordingly, dynamic postural control correlates with changes in occlusion both in subjects with craniomandibular dysfunctions and in the control group. [14]

This study confirmed interferences between postural control and the craniomandibular system in females aged between 41 and 50 years. There is a negative influence of angle class II malocclusion on postural control. In addition, when planning and fabricating dentures, a dental position in distal occlusion should be avoided in women between 41 and 50 years of age. Conversely, an interdisciplinary approach in dentistry could be applied to postural control disorders by functional analysis, so that possible limitations in lateral movements can be diagnosed and treated accordingly. In addition, weight-reduction measures are necessary as increasing weight and increased BMI have a negative effect on postural control. To what extent, however, these findings are relevant to everyday clinical practice, would have to be investigated in further studies. A comparison with other age groups would also be an interesting aspect at this point.

## Conclusion

In the case of subjectively healthy women between 41 and 50 years of age, an increase in weight and BMI leads to a shift in weight from the rearfoot to the forefoot. A limited laterotrusion to the right tends to result in a lower forefoot load and an increased rearfoot load. Laterotrusion to the left, which is above the norm, tends to show a lower frontal sway as well as a reduced elliptical area, height and width. Weak significant correlations regarding deviation and deflection were found. Thus, the extent of deviation correlates with reduced right forefoot loading, whilst the extent of deflection correlates with increased right foot loading and a reduced left foot loading. The higher the extent of angle class II malocclusion, the larger the ellipse area and height become. There are no significant correlations for midline shifts, transversal width differences or for crossbites and edge-to-edge bites.
